# Motion and form perception in healthy individuals with adolescent cannabis use

**DOI:** 10.3389/fpsyt.2026.1902131

**Published:** 2026-08-17

**Authors:** Balázs Barko, Szabolcs Kéri

**Affiliations:** 1 Sztárai Institute, University of Tokaj, Sárospatak, Hungary; 2 Department of Physiology, Albert Szent-Györgyi Medical School, University of Szeged, Szeged, Hungary

**Keywords:** adolescent, cannabis, development, motion perception, plasticity, vulnerability

## Abstract

**Introduction:**

Neuronal mechanisms of motion perception are susceptible to developmental anomalies. We tested whether heavy adolescent cannabis use is associated with enduring deficits in global motion perception, with relative sparing of global form perception, in abstinent adults.

**Methods:**

In a cross-sectional study, 130 adults were enrolled: cannabis-naïve controls (n=50), non-heavy adolescent users (n=38), and heavy adolescent users (daily use before age 15; n=42). Participants reported no current psychoactive substance use and were in sustained remission from cannabis-related problems for ≥12 months. DSM-5 psychiatric disorders were excluded. Motion coherence (random-dot display) and form coherence (orientation-defined circles) thresholds were measured. Cannabis exposure was quantified with the Cannabis Exposure Inventory (CEI).

**Results:**

The heavy-use group had higher motion coherence thresholds than controls and non-heavy users (*p* < 0.001; *BF_10_* > 10, strong Bayesian evidence). Form coherence thresholds did not differ between groups. Non-heavy users did not differ from controls on either task. The CEI scores predicted motion coherence thresholds.

**Conclusions:**

Heavy cannabis exposure starting in early adolescence is associated with a selective deficit in global motion integration, consistent with the hypothesis of increased vulnerability of the dorsal visual stream. This hypothesis should be confirmed by longitudinal studies.

## 1 Introduction

The use of cannabis during adolescence may have significant impacts on brain development and cognitive functions. Cannabis use during this period can disrupt the development of brain structures and functions, especially those related to memory, attention, and decision-making (1–5). Adolescents who use cannabis may experience impairments in various cognitive domains. Some studies suggest that these effects could be long-lasting, especially with heavy and prolonged use (4, 6, 7). There is evidence linking adolescent cannabis use with mental disorders, such as anxiety, depressive disorders, addictions, and psychosis (8–13). The risk is particularly high for individuals with a genetic predisposition to these conditions (14). Cannabis shows physiological interactions with the endocannabinoid system, which plays a role in neurodevelopment. Regular use during adolescence can lead to alterations in this system, causing long-term changes in brain function (15).

Certain cognitive impairments may be reversible after a period of abstinence. However, the extent of recovery may differ depending on the duration and intensity of cannabis use (7, 13, 16). Cannabis use during adolescence has been linked to deficits in both short-term and long-term memory (17–19). This effect is especially important because this developmental stage is critical for academic achievement and social skill learning (4). Adolescents using cannabis may also experience difficulties in focusing and maintaining attention. Attentional dysfunctions can manifest in academic settings as poor performance and difficulty in following complex tasks. Executive functions, which include planning, decision-making, problem-solving, and impulse control, can be impaired by cannabis use (20–24).

Much less is known about visual functions in early cannabis users. Adolescence is accompanied by continuing refinement of cortical inhibitory signaling, synaptic efficacy, and distributed network coordination. The endocannabinoid system participates in these processes (25). Endocannabinoids are synthesized on demand and act predominantly as retrograde signals at presynaptic cannabinoid type 1 (CB1) receptors, thereby regulating neurotransmitter release and activity-dependent plasticity. In the human and animal visual cortex, CB1 expression and other components of GABAergic signaling show marked age-related transitions extending through the teenage years (26, 27). Experimental studies further indicate that CB1-dependent signaling contributes to maturation of inhibitory transmission and experience-dependent plasticity in visual cortex (28, 29). Repeated exposure to tetrahydrocannabinol (THC) in adolescence therefore may perturb the development of visual circuits, although this remains a hypothesis rather than a demonstrated causal effect in humans.

The effect of adolescent cannabis use on the perception of shape and motion has not been studied so far, even though cannabis use is associated with several types of visual impairment (30–33). Cannabis impairs visual motion perception through multiple mechanisms, including deficits in motion coherence detection (30), smooth pursuit eye movements (34), tracking performance (35), and heading perception from optical flow (36). Critically, early-onset users display persistent visual scanning deficits that late-onset users do not, suggesting developmental vulnerability (37). Acute THC exposure increases motion coherence thresholds under all luminance conditions (30) and disrupts gamma-band neural oscillations during coherent motion perception (32). This indicates that cannabis exposure disrupts the brain’s ability to generate synchronized electrical activity required for complex visual processing. THC interferes with GABAergic and glutamatergic neurotransmission, the systems that control excitatory/inhibitory balance in the brain, and the consequent disruption of gamma synchrony may explain the altered perceptions and cognitive states often associated with cannabinoid use (32, 38, 39).

Most of the visual studies summarized above were conducted in adults who were current or regular cannabis users. Studies of early-onset users are more informative regarding developmental timing, but because visual performance was assessed in adulthood, they cannot determine when the difference emerged or whether it persisted continuously from adolescence. The present study addresses this gap by examining whether a history of heavy cannabis use beginning in early adolescence, as compared with non-heavy adolescent use, is associated with motion- and form-perception differences detectable in adulthood after sustained remission.

Visual functions offer a practical opportunity to detect developmental abnormalities at the behavioral level. For example, in schizophrenia, where cannabis use is a risk factor, there are neurodevelopmental alterations, including brain regions involved in visual processing (40–42). The ventral visual stream (occipital and inferior temporal regions) is implicated in form perception and object recognition, whereas the dorsal visual stream (occipital, parietal, and middle temporal cortex) is key for processing spatial location, motion, and attention (43–45). There is increasing evidence that various developmental disorders of the brain dominantly affect the dorsal visual stream, including Williams syndrome (46), autism-spectrum disorders (47–50), childhood hemiplegia (51), fragile X syndrome (52), developmental coordination disorder (53), developmental dyslexia (54, 55), cerebral visual impairment (56), amblyopia (57, 58), and transient visual deprivation (59). The dorsal cortical regions serving motion perception (V3a/accessory visual area 3 and V5/MT within the dorsal occipito-parietal and middle temporal visual pathways) may be particularly vulnerable to genetic and environmental influences affecting brain development and maturation (60–62). Atkinson (60) extended the dorsal visual stream vulnerability hypothesis to include dysfunctions in motion perception, visuomotor and spatial cognition, attentional processes, and numerical abilities.

These conditions are not presented as etiologically related to cannabis exposure. Rather, their relevance is methodological: they demonstrate that motion-sensitive visual functions may be disproportionately affected by diverse disturbances of brain development, thereby supporting the use of a motion–form comparison as a behavioral probe of developmental visual-system vulnerability. The cannabis-specific rationale for the present study derives from the preceding evidence on visual alterations in cannabis users and from the developmental role of cannabinoid signaling in visual circuits.

**Figure 1:**
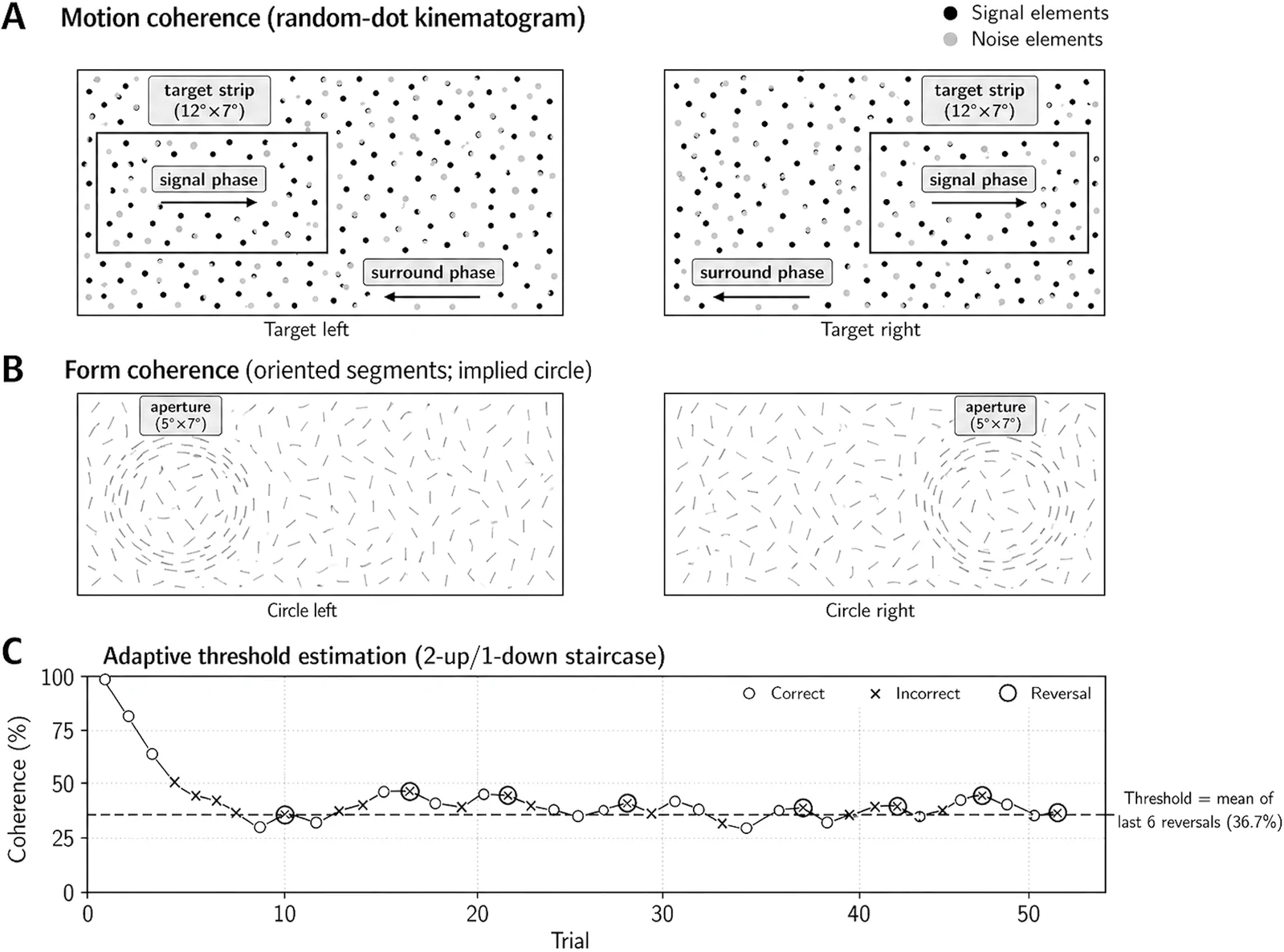
**(A)** *Motion coherence:* a random dot kinematogram contained a 12°×7° target strip presented on the left or right. Signal dots oscillated horizontally (6°/s) with direction reversals every 0.4 s; noise dots were randomly repositioned each frame. The target strip contained signal dots moving in opposite phase relative to the surrounding region. Coherence was defined as the ratio of signal dots to total dots. **(B)** *Form coherence:* within an aperture of 5° x 7°, located to the left or right of fixation, a proportion of 0.4°-line segments were tangentially oriented to form a circle. The remaining segments served as noise. **(C)** Coherence was adjusted adaptively with a 2-up/1-down staircase after an initial reduction. The threshold was defined as the mean of six reversals. Participants performed a left/right location judgment (keys 1/9).

The present study therefore addressed a specific comparison between two types of visual integration (63–65) (**Figure 1**). In the motion-coherence task, which relates to the dorsal stream, participants localized a strip defined by signal dots moving in counterphase with the surrounding field. The resulting threshold represents the minimum proportion of coherent motion signals required for motion-defined target–background segregation, and higher thresholds indicate reduced sensitivity. Normal motion coherence is essential in everyday situations such as detecting a pedestrian or cyclist in a busy road scene, walking through a crowded station or shopping area, or detecting an animal or person moving against visually complex surroundings.

In the form-coherence task, related to the ventral stream, participants localized a circle defined by tangentially oriented line segments embedded among randomly oriented noise elements. The form threshold therefore represents the minimum proportion of aligned orientation signals required for static contour integration, with higher thresholds again indicating reduced sensitivity. Form-coherence is essential for finding an object on a cluttered surface, recognizing a partially occluded object, and recognizing degraded signs and symbols.

Altogether, both tasks require extracting a structured signal from noise and estimating adaptive thresholds. The motion task, however, additionally depends on temporally precise integration of local motion signals and segregation of a dynamically defined target from its surround (63–65).

As mentioned before, previous studies have reported elevated motion-coherence thresholds in regular cannabis users and altered gamma-band neural activity during coherent-motion processing in heavy users (30, 32). Experimental cannabinoid administration also changes the temporal dynamics and population-level coordination of responses in early visual cortex (66). These observations led us to predict preferential alteration of motion-sensitive visual integration following heavy exposure beginning in early adolescence, consistent with the dorsal visual stream vulnerability hypothesis (60). Our primary hypothesis was that adults with a history of daily adolescent cannabis use would show higher motion-coherence thresholds than both cannabis-naïve controls and non-heavy adolescent users. Our specificity hypothesis was that the heavy-use group difference would be greater for motion coherence than for form coherence, yielding a group-by-task interaction. We further predicted that greater cannabis exposure would be associated with higher motion-coherence thresholds and that this association would be stronger than the corresponding association with form coherence. Because the study was cross-sectional, we examined whether these associations were detectable in adulthood after sustained remission rather than testing within-person persistence across development.

## 2 Materials and methods

### 2.1 Participants

In a cross-sectional, real-world study, we consecutively enrolled 130 participants via social media advertisements. The study was conducted between April 2024 and December 2025. The sample size was calculated to achieve 80% power (data analysis section). The advertisement stated: “We are looking for adults to take part in a study measuring how people perceive moving dots and visual patterns. You may be eligible if you never used cannabis or used cannabis occasionally as a teen or used cannabis daily before age 15; not currently using cannabis or other psychoactive substances; free of cannabis-related problems for at least 12 months; willing to complete screening questions (including mental health screening); and participation in a one-time session with computer-based visual tasks and questionnaires”.

In the recruited sample, 50 control individuals never used cannabis, 42 individuals were heavy users in adolescence (daily use before age of 15 years), and 38 individuals used cannabis only occasionally (non-heavy users). The participants did not receive any psychotropic medications and did not report other mental or neurological disorders. Participants showed sustained remission, which means that the criteria for cannabis use disorder or any other substance use disorder have not been met at any time for 12 months or longer (67). All participants had normal or corrected-to-normal visual acuity. The three groups were matched for age, sex, and socioeconomic status. The demographic parameters are shown in **Table 1**.

**Table 1:** Characteristics and comparison of the participants.

Characteristics	Controls (n=50)	Non-heavy cannabis user (n=38)	Heavy cannabis user (n=42)	*F*	*p*
Age (years)	31.2 (4.6)	30.5 (7.0)	29.6 (6.1)	0.89	0.41
Sex (male/female)	29/21	21/17	25/17	–	–
IQ	108.2 (8.1)	107.6 (10.6)	107.2 (10.9)	0.14	0.87
SES	31.0 (5.0)	31.8 (5.6)	29.8 (6.3)	1.38	0.25
BAI	6.3 (3.2)	6.5 (3.3)	8.6 (5.1)	4.69	0.01
BDI-II	9.8 (4.3)	9.1 (3.6)	11.6 (5.4)	3.28	0.04
CEI	–	47.4 (9.0)	127.7 (38.2)	159.7	< 0.001
EC-TLFB	–	8.9 (4.8)	8.4 (4.5)	0.25	0.62
Age at first cannabis use (years)	–	13.1 (0.9)	12.9 (1.5)	0.51	0.48
Duration of abstinence (months)	–	18.3 (4.9)	19.5 (5.2)	1.12	0.29
ASSIST alcohol	9.8 (4.7)	9.5 (4.6)	8.8 (4.7)	0.51	0.60
ASSIST other substances	2.9 (2.8)	20.6 (9.2)	20.4 (10.3)	77.09	< 0.001

SES, socioeconomic status; BAI, Beck Anxiety Inventory; BDI-II, Beck Depression Inventory-II; CEI, Cannabis Exposure Inventory (mgTHC/day); EC-TLFB, Enhanced Cannabis Timeline Followback (weekly THC units); ASSIST, Alcohol, Smoking and Substance Involvement Screening Test.

Data are mean (standard deviation), except for sex. CEI reflects adolescent exposure, whereas EC-TLFB reflects lifetime cannabis exposure.

The group means were compared with one-way ANOVA. The heavy user group scored higher than the non-heavy user and control groups on the BAI scale (*p* < 0.05, Tukey’s HSD test). The heavy user group scored higher than the non-heavy user group, but not higher than the controls, on the BDI-II scale (*p* < 0.05, Tukey’s HSD test). There was no significant difference between the heavy and non-heavy user group in ASSIST other substances scores (*p* = 0.94).

In summary, eligible participants were adults (age above 18 years) who were cannabis-naïve, had used cannabis occasionally during adolescence (non-heavy users), or had used cannabis daily before 15 years of age (heavy users) (duration of daily use: 7.8 years, SD = 3.1). All participants reported no current cannabis or other psychoactive substance use and had been in sustained remission from cannabis use disorder and any other substance use disorder for at least 12 months. Exclusion criteria were any DSM-5 psychiatric disorder, a self-reported neurological disorder, and current psychotropic-medication use. Psychiatric disorders were assessed using the SCID-5-CV administered by a trained clinician, as described in the previous sections.

Cannabis-use history was assessed separately in terms of developmental timing, frequency, duration, and estimated THC exposure. Cannabis-naïve controls reported no lifetime cannabis use. Non-heavy adolescent users reported cannabis exposure during adolescence but used cannabis at most once or twice per week and had never maintained a weekly or more frequent pattern. Early-onset heavy users reported daily use on 7 days per week/daily or near-daily use on at least 30 days beginning before their 15th birthday and continuing for several consecutive months. Participants reported consuming dried cannabis inflorescence (*Cannabis sativa* hybrid) with a mean total THC concentration of 18.5% and CBD smaller than 1.0%. They inhaled the product in a joint/pre-roll or via combustion in a water pipe (average: 0.5-0.8 grams/session). These consumption habits were similar in both user groups except for the frequency. None of the participants received treatment because of cannabis use disorder or other addictions.

We defined early onset as establishment of the relevant cannabis-use pattern before age 15. This threshold was selected *a priori* because previous studies have used cannabis exposure before age 15–16 to distinguish early- from later-onset use and have reported greater neurocognitive or attentional abnormalities in early-onset groups (37, 68). Human visual cortex also shows continuing developmental changes in CB1-related and GABAergic mechanisms during the teenage years (26).

### 2.2 Interviews and scales

We used the Structured Clinical Interview for DSM-5 Disorders—Clinician Version (SCID-5-CV) to assess the presence of mental disorders (69). A trained clinician administered the SCID-5-CV. We also used the Hungarian versions of the Beck Depression Inventory-II (BDI-II) (70) and the Beck Anxiety Inventory (BAI) (71) to characterize subclinical negative emotional experiences.

Socioeconomic status was assessed using the Hollingshead Four Factor Index, a composite measure based on educational attainment and occupational prestige, with marital and employment status. Scores range from 8 to 66, with higher values indicating higher socioeconomic status: 8–19 (unskilled laborers), 20–29 (semiskilled workers), 30–39 (skilled craftsmen, clerical, and sales workers), 40–54 (medium business, minor professional, and technical), and 55–66 (major business and professional) (72). General intellectual functions were measured with the Wechsler Adult Intelligence Scale-IV (WAIS-IV) (73). The clinical rating scales and interviews were administered by qualified raters who were blind to the aim of the study. Similarly, the assistants who administered the visual tasks were blind to the cannabis user status of the participants.

The participants completed the Cannabis Exposure Inventory (CEI) to characterize adolescent use, which is designed to measure cannabis use by assessing frequency, quantity (e.g., grams, puffs), product type (flower, concentrates), administration route, and estimated THC potency, aiming to standardize measurement. It includes questions, like time-of-day use patterns, and aims to calculate milligrams of THC per day (mgTHC/day) for reliable and comparable data distinguishing heavy and non-heavy users (74, 75) (**Table 1**). We also administered the Enhanced Cannabis Timeline Followback (EC-TLFB) to assess lifetime cannabis use, including standard THC units, which was validated through biological measures (76). Participants also completed the Alcohol, Smoking and Substance Involvement Screening Test (ASSIST), which separately evaluates lifetime alcohol and substance (e.g., tobacco, stimulants, hallucinogens) use (77). Following the initial screening, cannabis-use history was assessed using the CEI, EC-TLFB, ASSIST, and a clinician-administered chronological substance-use interview. The interview assessed age at first use, age at onset of weekly and daily use, duration of daily use, age at cessation, and periods of abstinence. Responses were compared across instruments, and discrepancies were clarified. Assessments were conducted by trained raters who were blind to the visual-task results and the directional study hypothesis. These procedures were intended to reduce social-desirability and eligibility-related biases. Current substance use was checked by a urine test (GC-MS, Abbott, Illinois).

### 2.3 Motion and form perception

The motion and form perception protocol followed our previously published procedure. Parts of the methodological description are adapted from that report to ensure accurate reporting of the identical task parameters (64) (**Figure 1**). Motion coherence was measured with random-dot stimuli in which only a subset of elements moved in a common direction while the remaining dots moved randomly. Participants were required to judge the direction of the global motion signal. Coherence was systematically varied by changing the proportion of dots moving coherently, allowing estimation of the minimum coherence level at which coherent motion could be reliably detected.

Form coherence was assessed using stimuli that differed in the degree to which their elements supported a recognizable shape or structure. Participants viewed degraded or fragmented forms and were asked to localize the represented object, contour, or configuration. In this context, coherence reflects the extent to which the visual elements are organized into a complete and interpretable form (64, 78).

Stimuli were presented on a ViewSonic PF815 monitor. The motion-coherence task used random-dot displays with a density of 4 dots/deg² and luminance of 85 cd/m². Signal dots moved horizontally at 6°/s and reversed direction every 0.4 s, whereas noise dots were randomly repositioned on each frame. Participants indicated whether the coherent motion strip appeared on the left or right side of the display. Thresholds were estimated with a 2-up/1-down staircase and calculated as the mean of six reversals.

In the form coherence task, stimuli were composed of 0.4°-line segments presented at a density of 19 segments/deg². Within an aperture of 5° x 7°, positioned either to the left or right of the screen center, a subset of line segments was oriented tangentially so as to define a circular form. The remaining line segments were randomly arranged and served as noise. Form coherence was determined by the proportion of tangentially aligned segments contributing to the circular contour. Participants indicated whether the circle appeared on the left or right side of the display (**Figure 1**).

For both tasks, the coherence level required for reliable detection was used as an index of visual sensitivity. Higher coherence thresholds indicate that a greater proportion of coherently moving dots or tangentially oriented line segments was needed for stimulus detection, reflecting reduced sensitivity of the visual system (63, 64, 79). Because the staircase terminated after six reversals, the number of trials varied across participants. The motion task comprised a mean of 26.4 trials (SD = 6.8), and the form task comprised a mean of 27.0 trials (SD = 5.9).

### 2.4 Data analysis

Data were analyzed using STATISTICA 13.1 (TIBCO) and JASP 0.96 (JASP Team, 2026). Descriptive statistics were first computed, and assumptions for parametric testing were evaluated using the Kolmogorov–Smirnov test for normality and Levene’s test for equality of variances. Coherence thresholds were then examined with analysis of variance (ANOVA). Group served as the between-subjects factor, with three levels: controls, participants with a history of heavy adolescent cannabis use, and participants with a history of non-heavy adolescent cannabis use. Task condition, defined as motion versus form coherence, was entered as the within-subjects factor. Effect sizes were reported as partial eta squared *(partial η²)*.

Where appropriate, significant effects were followed up using *F* tests (Holm-corrected) and Tukey’s Honestly Significant Difference (HSD) *post hoc* tests. Associations between coherence thresholds and clinical measures were assessed with Pearson product–moment correlation coefficients, with the False Discovery Rate procedure applied to control for multiple testing and reduce type I error. For correlations that remained significant after FDR correction, multiple regression analyses were additionally conducted.

An *a priori* power analysis was conducted using G*Power (3.1.9.7). The calculation targeted the group × task interaction in a mixed-design ANOVA with three between-subject groups and two within-subject task conditions. Assuming an interaction effect of Cohen’s f=0.14, corresponding to partial $\eta^{2}\approx 0.02$, a two-condition correlation of r=0.50, a nonsphericity correction of ϵ=1.00, an alpha level of 0.05, and statistical power of 0.80, the required total sample was 126 participants. The power calculation assumed equal group sizes, corresponding to 42 participants per group. The final sample included 130 participants (50 controls, 38 occasional users, and 42 heavy users), with modest deviation from equal group sizes reflecting consecutive recruitment and classification according to participants’ pre-existing cannabis-use histories.

Key between-group effects were further evaluated using Bayesian analyses in JASP 0.96. Bayes factors were interpreted as follows: BF_10_ values between 1 and 3 indicated weak evidence, values between 3 and 10 indicated moderate evidence, and values greater than 10 indicated strong evidence (64).

## 3 Results

### 3.1 Differences in motion and form perception

We first conducted a two-way ANOVA to examine differences among control individuals without cannabis use, non-heavy users, and heavy users in motion and form perception. This two-way ANOVA revealed significant main effects of group (*F*(2,127) = 5.20, *p* < 0.05; *partial η^2^* = 0.08) and task type (motion vs. form) (*F*(1,127) = 9.89, *p* < 0.005; *partial η^2^* = 0.07). The two-way interaction between group and task type was also significant (*F*(2,127) = 14.35, *p* < 0.001; *partial η^2^* = 0.18). However, planned comparisons with *F* tests revealed that the two-way interaction did not retain significance when the control and non-heavy user groups were compared (*p* = 0.74). The two-way interaction remained significant when the control and heavy user groups were compared (*F*(1,127) = 21.52, *p_Holm_* < 0.001, *partial η^2^* = 0.14), and when this comparison included the non-heavy user and heavy user groups (*F*(1,127) = 21.75, *p_Holm_*< 0.001, *partial η^2^* = 0.15).

**Figure 2:**
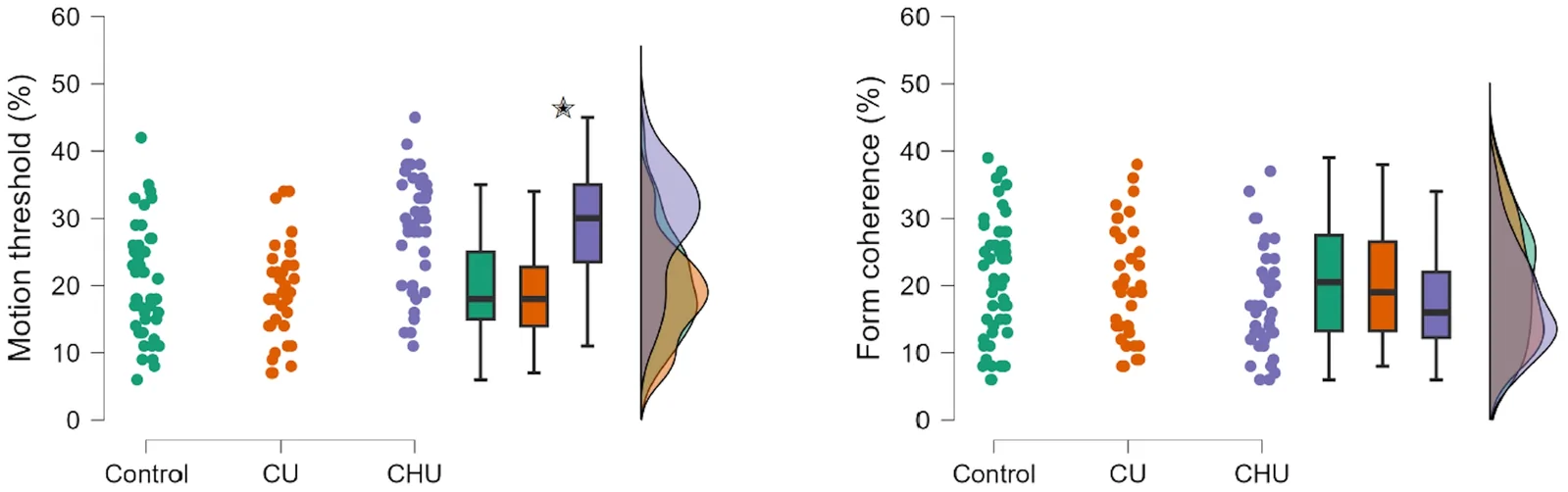
Results from the motion and form coherence tasks. Raincloud plots show the raw data points; median, quartiles (box), and spread of data (whiskers); data density (half-violin plots). Cannabis heavy users (CHU) showed higher motion thresholds relative to controls and non-heavy cannabis users (CU) (^*^p < 0.001, Tukey’s HSD tests).

Tukey’s HSD revealed higher motion coherence thresholds in the heavy user group relative to the non-heavy user and control groups (*p* < 0.001). However, there were no similar differences in form perception (*p* > 0.5). Moreover, the non-heavy user group did not differ significantly from the control group in the visual tasks (*p* > 0.5) (**Figure 2**; **Supplementary Table 1**).

### 3.2 Correlations between visual perception, demographic parameters, and clinical measures in cannabis users

When correlations between form perception and age, SES, IQ, BAI, BDI-II, CEI, EC-TLFB, and ASSIST scores were calculated, only one was significant, with IQ (*r* = 0.18, *p* < 0.05), but this significance was not retained after FDR correction. When the same correlations were calculated, including motion perception, only one was significant after FDR correction: CEI (*r* = 0.37, *p* < 0.001). Motion coherence threshold was predicted by CEI when the analysis was controlled for age, sex, SES, IQ, BAI, and BDI-II (*β* = 0.36, *SE* = 0.02, *p* < 0.001, *VIF* = 1.1). There were no significant correlations between form/motion thresholds and age at first cannabis use, duration of heavy use (years), and duration of abstinence (-0.2 < *rs* < 0.2). When these confounding variables were included in the ANOVAs as covariates, the results did not change. The full correlation matrix and the scatterplots are depicted in the Supplementary Material (**Supplementary Figure 1**; (**Supplementary Table 2**).

### 3.3 Bayesian analysis

To assess the strength of evidence for differences between the groups, we applied Bayesian t-tests to compare form and motion coherence thresholds. Under the null hypothesis, the standardized population mean difference was fixed at zero ( $H_{0}:\delta =0$). Under the alternative hypothesis, the standardized effect size was assigned a zero-centered Cauchy prior with scale $r=1/\sqrt{2}=0.707$. *BF_10_*denotes the ratio of the marginal likelihood of the data under the alternative hypothesis to that under the null hypothesis. There was no evidence that non-heavy users differed from controls in form and motion perception (form: *BF_10_* = 0.24, *error %* = 0.02; motion: *BF_10_* = 0.36, *error %* = 0.02). In contrast, there was strong evidence for a difference in motion perception between heavy users and controls (*BF_10_* = 4846.65, *error %* = 2.18 x 10^-10^), but not in form perception (*BF_10_*= 0.91, *error %* = 0.01). Importantly, when the non-heavy user and heavy user groups were compared, there was strong evidence for a between-group difference in motion perception (*BF_10_* = 77962.46, *error %* = 5.62 x 10^-8^), but not in form perception (*BF_10_*= 0.49, *error %* = 0.01).

## 4 Discussion

In this study, adults with a history of heavy cannabis use beginning early in adolescence showed a selective impairment in motion processing. Specifically, motion coherence thresholds were higher in heavy users than in both non-heavy users and cannabis-naïve controls. Form coherence was spared. Non-heavy cannabis use was not associated with changes in motion and form perception. However, greater cumulative cannabis exposure was associated with poorer motion sensitivity. This relationship was significant after controlling for demographic variables, IQ, and subclinical anxiety and depressive symptoms. Impaired motion processing in heavy adolescent cannabis users, relative to non-heavy adolescent cannabis users, cannot be explained by higher lifetime cannabis use, alcohol use, or the use of other psychoactive substances because heavy and non-heavy adolescent users did not show significant differences on these measures. The finding that heavy and non-heavy adolescent users markedly differed on the CEI scores (reflecting adolescent use) but were similar on the EC-TLFB scores (reflecting lifetime use) indicates that following the difference in adolescence, both user groups showed similar cannabis consumption habits.

Together, these findings are consistent with a lasting alteration in motion perception but not in form perception. The results fit well with the dorsal-stream vulnerability hypothesis, which proposes that motion-sensitive visual regions (MT/V5 and related occipito-parietal circuitry) are susceptible to developmental disturbances (60, 80). Accordingly, the present findings concern a visual difference observed in adulthood in association with adolescent-onset cannabis exposure, rather than a deficit measured directly during adolescence or demonstrated longitudinally to persist across development.

The present findings also extend the literature on cannabis and visual processing. In current cannabis users, Mikulskaya and Martin (2018) found increased motion coherence thresholds relative to non-users, together with lowered spatial contrast sensitivity under low luminance conditions (30). Similarly, Lalanne and colleagues (81) found that early-onset cannabis users showed reduced contrast sensitivity at low spatial frequencies, an effect that persisted after controlling attention and vigilance. Low spatial-frequency contrast sensitivity is frequently linked to dorsal visual stream functions (82).

Neurophysiological work also suggested a link between cannabis exposure and motion perception deficits. In heavy cannabis users, Skosnik and colleagues (32) observed reduced gamma-band power during coherent motion perception. However, early event-related potentials were spared, suggesting intact low-level sensory responses but altered cortical synchronization during motion perception. More recently, a magnetoencephalography (MEG) study suggested that gamma responses in the primary visual cortex are preserved in regular cannabis users, while functional connectivity involving higher-order regions is altered (39).

Other studies indicate that cannabis can influence both low- and high-level visual processing. Acute smoking has been associated with transient worsening across several visual parameters, including contrast sensitivity and night-vision (31). In addition, it has been linked to driving-relevant outcomes in simulator settings (83). Related evidence from the cognitive domain also suggests that cannabis may affect visuospatial functions. Ryder et al. (84) reported that acute cannabis intoxication selectively impaired visuospatial working memory, whereas auditory-verbal and short-term memory domains were relatively spared. Although that study examined working memory rather than visual motion perception, its findings may be relevant to the present results because both lines of work point to the importance of further examining cannabis-related vulnerability in visuospatial and spatial-processing systems.

A mechanistic interpretation of our findings is supported by the developmental regulation of cannabinoid signaling in the visual system. Cannabinoids are involved at multiple stages of visual processing, including retinal circuits and visual thalamocortical pathways, which supports the hypothesis that early cannabis use may lead to developmental visual dysfunctions (85). Autoradiographic mapping demonstrated cannabinoid receptor expression in the sensory regions (86). Importantly, CB1 cannabinoid receptor expression in human visual cortex exhibits developmental dynamics during childhood and adolescence (26, 87). Experimental work in primates further indicates that cannabinoid agonists can reduce neural responses in early visual cortex, as measured at the levels of EEG, local field potentials, and spiking activity (66). In this context, heavy exposure to THC during early adolescence, a period characterized by ongoing synaptic and circuit maturation, could bias the developmental trajectory of visual pathways. A dorsal-stream-selective effect is also biologically plausible because dorsal-stream functions show protracted development and may rely on precisely timed maturation of long-range connectivity and inhibitory-excitatory balance (60, 80).

However, it is notable that not all developmental findings are consistent. For example, prenatal cannabis exposure was associated with *better* global motion perception in preschool children, whereas prenatal alcohol exposure impaired motion perception (88). While this result cannot be directly compared with adolescent exposure, it highlights that developmental timing, task demands, and confounding factors may significantly affect the results. The authors emphasized that superior performance on this single task could not be extrapolated to beneficial fetal development or interpreted as “supernormal” neurodevelopment. In the Chakraborty et al. (88) study, a proportion of dots moved coherently upward or downward, whereas the remaining dots moved in random directions. The task in the present manuscript is computationally different. Participants localized a left- or right-sided strip whose signal dots moved in opposite phase to signal dots in the surrounding region. Successful performance therefore required not only integration of local motion signals within the target, but also maintenance of a spatial boundary, suppression of surrounding motion, and segregation of the target from a dynamically competing background.

Several limitations should be considered. First, the present study was cross-sectional, and cannabis exposure was assessed retrospectively. Although the dose–response association with CEI supports the role of exposure, it does not allow causal inference. The developmental interpretation is plausible, but the current design cannot determine whether cannabis exposure caused the perceptual differences, whether pre-existing visual or neurodevelopmental differences contributed to later cannabis-use patterns, or whether additional unmeasured factors were involved. Second, although participants were screened for psychiatric disorders, residual confounding remains possible (e.g., alcohol and smoking history, sleep quality, and subtle attentional differences). Third, the psychophysical tasks used here index specific types of motion and form perception. It remains unclear whether the deficit applies to other motion-perception tasks (e.g., radial motion, biological motion) and to real-world situations. Finally, participants were aware from the online advertisement that cannabis-use history was relevant to the study, and this could introduce some degree of self-selection or expectancy effects.

Further research is needed, including longitudinal approaches with repeated measurements throughout adolescence and early adulthood. The role of the dorsal-stream circuitry should be examined using psychophysics, neuroimaging, and electrophysiology. Lastly, since many moderators may affect task performance (e.g., age of onset, use of nicotine or alcohol, type of cannabinoids, THC-dominant vs. balanced THC/CBD products), it would be essential to assess these factors. Functional outcomes in daily life are also of special importance (e.g., driving-relevant demands, subjective visual symptoms, and hazard perception).

## 5 Conclusions

The results suggest that heavy adolescent cannabis use may be associated with a specific impairment of motion perception, whereas form perception remains intact. Moreover, worse motion sensitivity was predicted by greater cumulative exposure to cannabis. This evidence supports the hypothesis that there are lasting deficits in the functions of the dorsal visual stream, which may be associated with earlier heavy cannabis use and its developmental effects. These findings should be replicated by longitudinal investigations. At the community level, these findings underscore the need for adolescent-focused prevention, education, and early intervention efforts that address potential neurodevelopmental risks of heavy cannabis use.

## Data Availability

The raw data supporting the conclusions of this article will be made available by the authors, without undue reservation.
