# Supplementary material for: Motion and form perception in healthy individuals with adolescent cannabis use
Source: Front Psychiatry. 2026 Aug 17;17:1902131. doi: 10.3389/fpsyt.2026.1902131 (PMC13526967; doi:10.3389/fpsyt.2026.1902131)
Supplement: Supplementary file 1 [file Supplementaryfile1.docx]

Supplementary Material

# Supplementary Figures and Tables

|  | | | | | | |
| --- | --- | --- | --- | --- | --- | --- |
|  | **Motion** | | | **Form** | | |
|  | controls | non-heavy  user | heavy  user | controls | non-heavy  user | heavy  user |
| n | 50 | 38 | 42 | 50 | 38 | 42 |
| Mean | 20.34 | 18.66 | 28.76 | 20.60 | 19.79 | 17.43 |
| 95% CI A. Mean Lower | 18.09 | 16.29 | 26.17 | 18.04 | 17.00 | 15.06 |
| 95% CI A. Mean Upper | 22.59 | 21.03 | 31.36 | 23.16 | 22.58 | 19.80 |
| Std. Deviation | 7.901 | 7.212 | 8.325 | 8.992 | 8.479 | 7.597 |
| **Pairwise comparison** | **Motion: Hedges' g (95% CI)** | | | **Form: Hedges' g (95% CI)** | | |
| Non-heavy users vs. controls | -0.22 [-0.64, 0.20] | | | -0.09 [-0.51, 0.33] | | |
| Heavy users vs. controls | 1.03 [0.59, 1.47] | | | -0.37 [-0.79, 0.04] | | |
| Heavy users vs. non-heavy users | 1.28 [0.80, 1.76] | | | -0.29 [-0.73, 0.15] | | |

**Supplementary Table 1.** Descriptive statistics and pairwise standardized effect sizes from the form and motion coherence tasks.

**Correlation matrix of the variables**

| *Pearson's Correlations* | | | | | | | | | | | | | | | | | | | |
| --- | --- | --- | --- | --- | --- | --- | --- | --- | --- | --- | --- | --- | --- | --- | --- | --- | --- | --- | --- |
| Variable | |  | | Motion | | Form | | age | | SES | | IQ | | CEI | | BAI | | BDI2 | |
| 1. Motion |  | Pearson's r |  | — |  |  |  |  |  |  |  |  |  |  |  |  |  |  |  |
|  |  | p-value |  | — |  |  |  |  |  |  |  |  |  |  |  |  |  |  |  |
| 2. Form |  | Pearson's r |  | -0.138 |  | — |  |  |  |  |  |  |  |  |  |  |  |  |  |
|  |  | p-value |  | 0.116 |  | — |  |  |  |  |  |  |  |  |  |  |  |  |  |
| 3. age |  | Pearson's r |  | 0.148 |  | -0.147 |  | — |  |  |  |  |  |  |  |  |  |  |  |
|  |  | p-value |  | 0.092 |  | 0.094 |  | — |  |  |  |  |  |  |  |  |  |  |  |
| 4. SES |  | Pearson's r |  | -0.098 |  | 0.019 |  | 0.086 |  | — |  |  |  |  |  |  |  |  |  |
|  |  | p-value |  | 0.266 |  | 0.828 |  | 0.329 |  | — |  |  |  |  |  |  |  |  |  |
| 5. IQ |  | Pearson's r |  | 0.006 |  | 0.182 | * | -0.087 |  | -0.148 |  | — |  |  |  |  |  |  |  |
|  |  | p-value |  | 0.943 |  | 0.038 |  | 0.322 |  | 0.092 |  | — |  |  |  |  |  |  |  |
| 6. CEI |  | Pearson's r |  | 0.373 | *** | -0.181 |  | -0.158 |  | -0.112 |  | -0.050 |  | — |  |  |  |  |  |
|  |  | p-value |  | <.001 |  | 0.108 |  | 0.162 |  | 0.324 |  | 0.663 |  | — |  |  |  |  |  |
| 7. BAI |  | Pearson's r |  | 0.215 | * | -0.098 |  | -0.040 |  | -0.047 |  | -0.054 |  | 0.100 |  | — |  |  |  |
|  |  | p-value |  | 0.014 |  | 0.266 |  | 0.651 |  | 0.599 |  | 0.542 |  | 0.379 |  | — |  |  |  |
| 8. BDI2 |  | Pearson's r |  | 0.086 |  | 0.003 |  | 0.004 |  | -0.064 |  | 0.032 |  | 0.128 |  | 0.140 |  | — |  |
|  |  | p-value |  | 0.328 |  | 0.972 |  | 0.965 |  | 0.467 |  | 0.714 |  | 0.258 |  | 0.111 |  | — |  |
|  | | | | | | | | | | | | | | | | | | | |
| * p < .05, ** p < .01, *** p < .001 (uncorrected values) | | | | | | | | | | | | | | | | | | | |

**Supplementary Table 2.** Correlation matrix of variables.

**Supplementary Figure 1.** Scatterplot of the correlation matrix showing the raw data.

BAI – Beck Anxiety Inventory, BDI-II – Beck Depression Inventory-II CEI - Cannabis Exposure Inventory, SES – socioeconomic status
